# A profile of adult acute admissions to Lentegeur Psychiatric Hospital, South Africa

**DOI:** 10.4102/sajpsychiatry.v24i0.1282

**Published:** 2018-09-27

**Authors:** Herman Franken, Robert Wicomb, Robin Allen, John Parker

**Affiliations:** 1 Department of Psychiatry, Faculty of Medicine and Health Sciences, Stellenbosch University, South Africa; 2 Department of Psychiatry and Mental Health, Faculty of Health Sciences, University of Cape Town, South Africa; 3 Lentegeur Hospital, South Africa; 4 Capital and Coast District Health Board, New Zealand; 5 The Royal Australian and New Zealand College of Psychiatrists, New Zealand

## Abstract

**Introduction:**

The Western Cape Province has the highest documented lifetime prevalence of common mental disorders in South Africa. To ensure the efficient, equitable and effective distribution of current resources, there is a need to determine the profile of patients seeking psychiatric treatment.

**Aim:**

The aim of this study was to describe patients admitted to the Acute Adult Admissions Unit at Lentegeur Hospital (LGH) which is situated in Mitchells Plain, Cape Town.

**Methods:**

This retrospective study involved an audit of all patients (18–60 years of age) admitted between 01 January 2016 and 30 June 2016.

**Results:**

A total of 573 adult psychiatric patients were profiled. The median age of the cohort was 29 years. The majority of patients (63%) were educated to the secondary level. Only 12% of patients were employed and 37% received disability grants. More than 90% of cases presented with psychotic symptoms. Of these, 28% presented with first episode psychosis. Of all patients, 20% were referred with manic symptoms and 7% with depressive symptoms. Many patients (62%) used substances in the period leading up to admission. Significantly more males (73%) used substances compared to females. Cannabis was the most widely used substance (51%), followed by methamphetamine (36%). Violence was a contributing factor to 37% of admissions. A total of 70 patients (13%) tested positive for HIV, while 49 (9%) tested positive for syphilis.

**Conclusion:**

We found the average patient requiring admission to be a young urban male who would likely have a secondary level (grades 8–12) education, but be unemployed. This patient would also likely be a user of illicit substances, would present psychotic and would likely display violent behaviour prior to referral. Substance use and a propensity for violence were identified as significant factors that influence the likelihood of admission. We invite planners in the Department of Health, as well as other stakeholders, to take heed of this burgeoning crisis and to implement specific strategies for addressing these problems before the effectiveness of mental health services as a whole is further undermined.

